# Treatment of Diabetes Mellitus

**Published:** 1852-12

**Authors:** 

**Affiliations:** Paris


*Treatment of Diabetes Mellitus.* By M. TROUSSEAU, of Paris.

The following advice, given by M. Trousseau, in writing to a patient affected with diabetes mellitus, will, we think, interest our readers. The patient, a female, aged about fifty, had suffered from the disease for several years, but had received no systematic treatment until she consulted M. Trousseau, visiting Paris for that purpose. The treatment directed by that distinguished physician has, thus far, had a signally good effect. The quantity of urine is much diminished, although it still contains sugar; the thirst has ceased to be troublesome, and she has gained in weight and strength. It will be observed that but little more than two months have elapsed since the date of the consultation. The local affection (pruritus with ecrema) prior to consulting M. Trousseau was intolerable. It ceased in a few days after resorting to the remedies prescribed, and has not since returned.

" *Consultation.*

"The almost unquenchable thirst which *Madame* feels; the extreme abundance of the urine; the general debility; the pruritus and chronic ecrema f the extreme genital parts, led me to suspect the existence of *diabetes mellitus;* and the chemical analysis of the urine places its existence beyond a doubt. The urine is much heavier than in the normal state, and contains a large amount of *glucose.* The disease has already existed for a long number of years, and has become in some measure constitutional, so that its complete cure will be very difficult, and it will be likely to return often if the patient will not submit to a suitable regimen, and take, from time to time, the remedies which I am about to indicate.

"It will be doubtless easy to arrest the production of sugar, by giving only articles of food taken from the animal kingdom, thus depriving the disease of every thing which contains sugar or starch; but in that manner the recovery will not be complete, and I hope far greater benefit from a regimen altogether different from that just stated, and a course of medication which I will indicate.

" *Treatment.*

"During the four months following, *Madame* will take, in the water which she drinks, about a drachm, gradually increasing to half an ounce, of the bicarbonate of soda. If it cause diarrhœa, she will omit the solution of the bicarbonate of soda, and take, at meals, lime-water, in doses of one, two, or even four ounces, twice a day. If, on the contrary, there is too obstinate constipation, do not omit the bicarbonate of soda, but twice a week let her purge herself with milk of magnesia.

"During the winter, if the skin be dry, *Madame* will take, in her drink, each day, from one to four *grains* of phosphate of ammonia.

"When the alkaline regimen has been followed conscientiously for four months, it is to be omitted for a month — again followed for two months — omitted again for six weeks or two months, and afterward followed each three months for a month at a time.

"Meanwhile the diet which ought to be adopted plays an important part in the treatment, and is to be modified in the following manner: very little bread — few potatoes — little rice — and, in general, little farinaceous substances. I say little, for I do not exclude them altogether from the diet.

"Substitute as much as possible in the food, green vegetables, such as new small peas, green beans, sorrel, chiccory, salad, artichokes. Eat considerable fruit, such as strawberries, raspberries, currants, cherries, pears and apples, but not sweet ones.

"Very little of meats (*viandes*) in general. However, when *Madame* is using the alkaline remedies in large doses she may eat a greater amount of meat. On the contrary when she intermits the alkaline she must diminish very much the proportion of meats.

"The rapid and very decided relief which the disease undergoes under the use of the alkaline medication, may lead one to exaggerate their use, and I am not certain but that if these therapeutical agents were too perseveringly given, they might cause an accident worse than diabetes. But I again repeat here, that the interrupted treatment which I have indicated, may be continued for a very great number of years.

"It remains for me to indicate the proper method to combat the ecrema of the genital parts, and the pruritus which accompanies it. Although this affection may be produced by the constant irritation of altered urine, it may persist when the urine returns to its normal condition. To relieve this disease, I propose the following means:

"For the *particular toilet* use every morning and evening water as warm as can be borne. Warm water suffices sometimes. If it be insufficient, add a solution, in alcohol, of corrosive sublimate.

℞ Sublimate corrosive, ʒii.

Alcohol, ℥iv.

Solve.

"Use four teaspoonfuls of this solution in four pints of very warm water, for bathing and for injecting.

"Substitute for the sublimate, solutions of sulphate of zinc, sulphate of copper, borax, the oleo-calcareous liniment, lime-water, tar-water, spirits of turpentine, etc. etc.

"When the pruritus diminishes, the warm water should be quitted by degrees, not suddenly. Notwithstanding the remedies I have indicated, I am persuaded that the malady will return if the warm water be left off too suddenly.

"Although the advice I have given differs, in many respects, from the ordinary course of treatment of *glucosurie*, I do not the less persist, because considerable experience has convinced me of the utility of this plan of medication.

A. TROUSSEAU,

Paris, Sept. 8, 1852. 52 Rue Café du Rampart."

